# Effects of transcriptional mode on promoter substitution and tandem engineering for the production of epothilones in *Myxococcus xanthus*

**DOI:** 10.1007/s00253-018-9023-4

**Published:** 2018-04-28

**Authors:** Xin-jing Yue, Xiao-wen Cui, Zheng Zhang, Wei-feng Hu, Zhi-feng Li, You-ming Zhang, Yue-zhong Li

**Affiliations:** State Key Laboratory of Microbial Technology, School of Life Science, Shandong University, Jinan, 250100 China

**Keywords:** Promoter, Substitution, Tandem engineering, Transcription mode, Epothilone biosynthesis, *Myxococcus xanthus*

## Abstract

Promoter optimization is an economical and effective approach to overexpress heterologous genes and improve the biosynthesis of valuable products. In this study, we swapped the original promoter of the epothilone biosynthetic gene cluster in *Myxococcus xanthus* with two endogenous strong promoters P_*pilA*_ and P_*groEL1*_, respectively, which, however, decreased the epothilone production ability. The transcriptional abilities by the two promoters were found to be bloomed in the growth stage but markedly decreased after the growth, whereas the original promoter P_*epo*_ functioned majorly after the exponential growth stage. Tandem repeat engineering on the original promoter P_*epo*_ remarkably increased epothilone production. The tandem promoter exerted similar expressional pattern as P_*epo*_ did in *M. xanthus*. We demonstrated that differential transcriptional modes markedly affected the efficiency of promoters in controlling the gene expressions for the production of the secondary metabolite epothilones. Our study provides an insight into exploiting powerful promoters to produce valuable secondary metabolites, especially in host with limited known promoters.

**Electronic supplementary material:**

The online version of this article (10.1007/s00253-018-9023-4) contains supplementary material, which is available to authorized users.

## Introduction

The gene transcriptional regulation is orchestrated by multiple molecular mechanisms (Browning and Busby 2016; Splinter and de Laat 2011). RNA polymerase recognizes and binds to the promoter DNA sequence to initiate the gene transcription. Promoter is thus the central element for regulating gene transcriptions (Browning and Busby 2004; Roy and Singer 2015). Using a promoter that is efficient in host is a common strategy to overexpress heterologous genes and thus improve the product yields. Hence, optimization of promoter has long been regarded as an efficient approach in the regulation of gene expressions (Terpe 2006), which is usually evaluated by the final expression of a reporter gene or a product (Luo et al. 2015; Zhu et al. 2015a). Many efforts have been performed on promoters, such as promoter replacement (Qiu et al. 2014), mutation (Alper et al. 2005), and engineering of regulatory elements of promoters (Liu et al. 2008), to generate an efficient promoter system. However, the transcriptional mode and its effects on promoter engineering have been less investigated.

Epothilones are a kind of polyketide compounds with the anticancer mechanism mimicking Taxol and have attracted great attention by the high activity towards P-glycoprotein-expressing multidrug-resistant tumor cell lines (Bollag et al. 1995; Vichai and Kirtikara 2006). Up to now, more than six epothilones or their derivatives are being evaluated in different stages of clinical trials, and ixabepilone has been approved for the clinical treatment of advanced breast cancer by the Food and Drug Administration of the USA (Brogdon et al. 2014; Huang et al. 2010; Rivera et al. 2008; Roche et al. 2007). Epothilones are originally produced by the myxobacterium *Sorangium cellulosum* (Gerth et al. 1996), which has a long doubling time and limited molecular performance tools. To efficiently produce epothilones, researchers have heterologously expressed the epothilone biosynthetic gene cluster in different hosts, including *Myxococcus xanthus*, the model species of myxobacteria (Bian et al. 2017; Fu et al. 2008; Julien and Shah 2002; Müller 2009; Mutka et al. 2006; Osswald et al. 2014; Park et al. 2008; Tang et al. 2000). Although *M. xanthus* is rather fermentation-friendly and easily genetic manipulated, the heterologous production of epothilones in *M. xanthus* is not yet superior to that in *S. cellulosum* and still far from meeting the demand of industrial production. For overexpressing target genes, Fu et al. once replaced the original promoter with an efficient promoter Tn5 for the heterologous biosynthesis of epothilones, which, however, yielded fewer amounts of products (Fu et al. 2008). Until now, engineering on the promoter for the biosynthesis of epothilones has been less investigated. The information on how to select and engineer an efficient promoter to improve the epothilone production is still lacking.

**Fig. 1:**
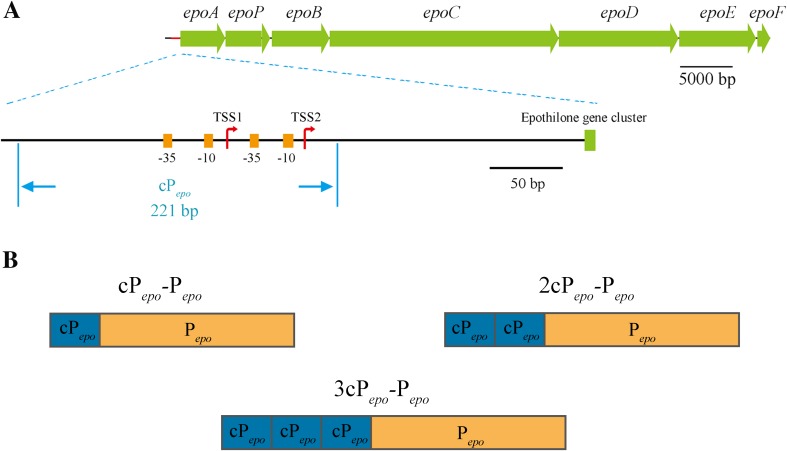
**a** Physical map of the epothilone biosynthetic gene cluster and the promoter P_*epo*_. TSS, transcription starting site; − 10, − 10 region; − 35, − 35 region. **b** Physical map of the tandem promoters

In our previous studies, the whole gene cluster for the biosynthesis of epothilones, including the promoter sequence (Fig. 1a), was integrated into the *M. xanthus* genome by transposition insertion (Zhu et al. 2015b). In this paper, we substituted the original promoter with strong endogenous promoters and made tandem repeat engineering on the original promoter to evaluate their effects on the production of epothilones in *M. xanthus*. We investigated and discussed the transcriptional mode of different promoters and their effects on promoter engineering for the production of epothilones.

## Materials and methods

### Bacterial strains, plasmids, and culture conditions

*Escherichia coli* DH5α was used as the host for pBJ113 plasmid construction, and *E. coli* DH5α *λ pir* was used for pSWU19 plasmid. *E. coli* strains were grown at 37 °C in Luria-Broth (LB) medium (10 g/L peptone, 5 g/L yeast extract, and 5 g/L NaCl, pH 7.2).

The *M. xanthus* laboratory strains DK1622 and DZ2 (Müller et al. 2013) are both originated from the wide-type strain *M. xanthus* FB (ATCC 19368). These two strains are widely used as model strains in fundamental researches of myxobacteria. *M. xanthus* ZE10 was constructed in our previous work by integrating the epothilone biosynthetic gene cluster of So0157-2 (CCTCC M 208078) into the genome of *M. xanthus* DZ2 (Zhu et al. 2015b). *M. xanthus* strains were grown at 30 °C in CYE medium [10 g/L casitone, 5 g/L yeast extract, 10 mM 3-(N-morpholino) propanesulfonic acid (MOPS), and 4 mM MgSO_4_, pH 7.6] or CMO medium [10 g/L casitone, 5 g/L yeast extract, 10 mM MOPS, 4 mM MgSO_4_, and 7 mL/L methyl oleate, pH 7.6]. The medium was supplemented with the following antibiotics if required: kanamycin [Km] 40 μg/mL; apramycin [Apra] 30 μg/mL.

Bacterial strains and plasmids used in this study are listed in Table S1.

### Construction of cP_*epo*_ promoter fragments

p15A-CT-epo, a plasmid containing the epothilone gene cluster and some flanking sequences, was constructed in our previous work (Zhu et al. 2015b). We obtained three cP_*epo*_ promoter fragments with different restriction enzyme cutting sites by PCR using three pairs of primers (P1-F/P1-R, P2-F/P2-R, and P3-F/P3-R) with p15A-CT-epo as template. All the fragments were cloned into pBJ113 at the corresponding restriction enzyme cutting sites, resulting in pBJ113-3cP_*epo*_, which were verified by enzyme-cutting and sequencing with primers M13-F and M13-R. Then, the pBJ113-3cP_*epo*_ plasmid was digested with the enzyme pairs of *Kpn*I/*Xma*I, *Kpn*I/*Bam*HI, and *Kpn*I/*Xba*I, respectively, to obtain three DNA fragments, named 1cP_*epo*_, 2cP_*epo*_, and 3cP_*epo*_. The tandem promoter clusters are shown in Fig. 1b.

### Construction of promoter vectors

Using pJBA28 (Andersen et al. 1998) and p15A-CT-epo as templates, the *gfp* gene and the original epothilone promoter fragment P_*epo*_ were amplified by PCR with primers *gfp*-F1/*gfp*-R1 and pE-F/pE-R, respectively, and then ligated by overlap extension PCR to obtain P_*epo*_-*gfp*. The fusion fragment P_*epo*_-*gfp* was cloned into the *Xba*I/*Hin*dIII sites of pSWU19, resulting in pSWU19-P_*epo*_-*gfp*. Besides, the *gfp*2 fragment, containing the same *gfp* gene sequence but different flanking restriction enzyme cutting sites, was amplified by PCR with primers *gfp*-F2/*gfp*-R2 and inserted into the *Hin*dIII/*Xba*I sites of pSWU19, resulting in pSWU19-*gfp*. The P_*pilA*_ and P_*groEL1*_ fragments were obtained by PCR using primers P_*pilA*_-F/P_*pilA*_-R and P_*groEL1*_-F/P_*groEL1*_-R with the genome of *M. xanthus* DK1622 as template and then cloned into the *Xba*I/*Kpn*I sites of pSWU19-*gfp*, leading to pSWU19-P_*pilA*_-*gfp* and pSWU19-P_*groEL1*_-*gfp*, respectively. The diagrammatic sketch for the reporter vectors is shown in Fig. S1. The 1cP_*epo*_, 2cP_*epo*_ and 3cP_*epo*_ fragments were inserted into the different sites of pSWU19-P_*epo*_-*gfp* to construct plasmids pSWU19-1cP_*epo*_-P_*epo*_-*gfp*, pSWU19-2cP_*epo*_-P_*epo*_-*gfp* and pSWU19-3cP_*epo*_-P_*epo*_-*gfp*, respectively (shown in Fig. S2). The pSWU19-F/pSWU19-R primers were used for sequencing of the constructed plasmids. All recombinant plasmids with *gfp* were verified by enzyme-cutting and sequencing.

To construct the plasmids used in *M. xanthus* to manipulate epothilone promoter, two pairs of up-arms and down-arms for homologous recombination were amplified using primers L1-F/L1-R, R1-F/R1-R, and L2-F/L2-R, R2-F/R2-R with p15A-CT-epo as template. The up-arm1 and down-arm1 were inserted into the *Eco*RI/*Kpn*I and *Xba*I/*Hin*dIII sites of plasmid pBJ113, respectively, resulting in pBJ113-L_1_R_1_. With up-arm2 and down-arm2 inserted into pBJ113, pBJ113-L_2_R_2_ was constructed similarly. The promoter fragments 3cP_*epo*_ was cloned into the *Kpn*I/*Xba*I sites of pBJ113-L_1_R_1_ to obtain the plasmid pBJ113-L_1_R_1_-3cP_*epo*_. P_*pilA*_ and P_*groEL1*_ were separately introduced into the *Kpn*I/*Xba*I sites of pBJ113-L_2_R_2_ to obtain the plasmids pBJ113-L_2_R_2_-P_*pilA*_ and pBJ113-L_2_R_2_-P_*groEL1*_.

The primers used in plasmids construction are listed in Table S2.

### Fluorescence assay in *E. coli* and *M. xanthus*

The *gfp*-carrying recombinant pSWU19-derived plasmids were transformed into *E. coli* DH5α by transformation. Cells harboring different promoters for *gfp* expression were grown in 50 mL LB medium (Km) at 200 rpm and 37 °C overnight, and then transformed into 50 mL LB medium (Km) by 2% inoculum size. After 24 h of incubation, cells were harvested by centrifugation at 12,000×*g* for 1 min and resuspended with PBS buffer (137 mM NaCl, 2.7 mM KCl, 10 mM Na_2_HPO_4_, 2 mM KH_2_PO_4_, pH 7.4). Two hundred microliters of the suspension was transferred into a 96-well plate in which OD_600_, and fluorescence was read with excitation at 485 nm and emission at 526 nm using an EnSpire™ Multimode Plate Reader (U.S.A).

Plasmids carrying *gfp* derived from pSWU19 were introduced into *M. xanthus* DZ2 by electrotransformation as described previously (Zhu et al. 2015b). The resulting strains were grown in 50 mL CYE liquid medium with Km at 30 °C for 20 h and then inoculated to 50 mL CMO medium containing 2% of the XAD-16 resin at a ratio of 1:50. The strains were sampled at four measurement points (12, 24, 36, and 48 h of incubation). Then, the OD_600_ and fluorescence of samples were analyzed by a UNICO-7200™ Spectrophotometer (U.S.A) and a F-4600™ Fluorescence Spectrophotometer (Japan), respectively.

### Construction of promoter manipulation mutants

The recombinant plasmids pBJ113-L_1_R_1_-3cP_*epo*_, pBJ113-L_2_R_2_-P_*pilA*_, and pBJ113-L_2_R_2_-P_*groEL1*_ were introduced into ZE10 by electrotransformation as we reported previously (Zhu et al. 2015b). Resistant colonies that appeared after 6 days of incubation on CYE plates with 40 μg/mL of Km were checked by colony PCR with primers KG-F and KG-R. The Km-resistant colonies were resuspended with CYE medium, mixed with 2.5 mL of CYE medium containing 0.5% soft agar, and spread on CYE plates containing 0.1% galactose to screen the second homologous recombinants. The galactose-resistant but Km-sensitive colonies were selected and checked by colony PCR with primers Test-F/Test-R and L2-F/R2-R. The mutant strains, named ZE10-3cP_*epo*_, ZE10-A-P_*pilA*_, and ZE10-A-P_*groEL1*_, were confirmed by sequencing of the PCR products. The primers used in construction of mutants are listed in Table S2.

### Extraction and detection of epothilones

The extraction and detection of the production of epothilones in *M. xanthus* recombinants were performed according to our previously reported methods (Zhu et al. 2015b). Briefly, ZE10, ZE10-3cP_*epo*_, ZE10-A-P_*pilA*_, and ZE10-A-P_*groEL1*_ were cultured in 50 mL CYE liquid medium overnight and then inoculated into 50 mL CMO medium containing 2% of the XAD-16 resin at a ratio of 1:50. After 6 days of incubation, the resin was collected into the 10-mL centrifuge tubes in which 3 mL methanol was added to extract the epothilones overnight. The leaching liquor was filtered with 0.22-μm filter and analyzed by high-performance liquid chromatography (HPLC) (SHIMADZU, Japan) monitored at 249 nm. The analytes were eluted at a flow rate of 1.0 mL/min, with mobile phase of 60% of methanol and 40% of H_2_O. The yields of epothilones were quantified based on the peak area in the UV chromatogram.

### Transcriptional analysis of epothilone genes

We collected samples continuously from the fermentation culture at four measurement points (12, 24, 36, and 48 h of incubation). Then, total RNA of samples was extracted using BIOZOL kits (Total RNA Extraction Regent, BioFast, China) and then transcribed reversely into cDNA with PrimeScript™ Regent Kit with DNAase (Takara, Japan). The *gapA* gene (glyceraldehyde-3-phosphate dehydrogenase gene, MXAN_2815) was chosen as the reference gene for normalization. The transcriptional level of epothilone gene cluster was analyzed by RT-qPCR on LightCycler^®^ 480 (Switzerland) with SYBR^®^ Premix Ex Taq™ GC Dye (Takara, Japan). All the primers used in RT-qPCR are listed in Table S3.

### Statistical analysis

The difference significance was analyzed statistically by using the IBM SPSS Statistics for independent-samples *t* - test.

## Results

### Selection of endogenous promoters and their transcriptional strengths

P_*epo*_ is the original promoter of the epothilone gene cluster. The promoter with the biosynthetic gene cluster from *S. cellulosum* So0157-2 (Han et al. 2013) was introduced by transposition insertion into the *M. xanthus* genome (Zhu et al. 2015b). The production of epothilones controlled by this original promoter in *M. xanthus* is normally less than 1 mg/L, which was further improved by approximately ten times with the supplementation of methyl oleate in the fermentation medium (Yue et al. 2017). To replace the original promoter with a stronger promoter for higher production, we chose two promoters originated from *M. xanthus* for the promoter-swapping experiment, the promoter of the *pilA* gene (P_*pilA*_), and the promoter of the *groEL1* gene (P_*groEL1*_). The *pilA* gene (MXAN_5783) encodes the subunit of the major pilin protein (PilA) for the constitution of type IV pili of *M. xanthus* (Craig et al. 2004). The promoter of the *pilA* gene is a σ54 promoter and is active during the vegetable growth and development stages (Wu and Kaiser 1997). P_*pilA*_ has been often used as a strong promoter to overexpress target genes in *M. xanthus* (Jakovljevic et al. 2008; Peng et al. 2017). The *groEL1* gene (MXAN_4895) encodes a type I chaperonin involving in many cellular processes (Kerner et al. 2005). Similarly, the *groEL1* is expressed at a high level in conventional cultivation (Wang et al. 2013; Zhuo et al. 2017). The brief information of these three promoters is listed in Table 1.

**Table 1:** Brief information about the studied promoters

Promoter	Gene	Length	Source
P_*pilA*_	*pilA*	630 bp	*M. xanthus* DK1622
P_g*roEL1*_	*groEL1*	500 bp	*M. xanthus* DK1622
P_*epo*_	Epothilone gene cluster	840 bp	*S. cellulosum* So0157-2

**Fig. 2:**
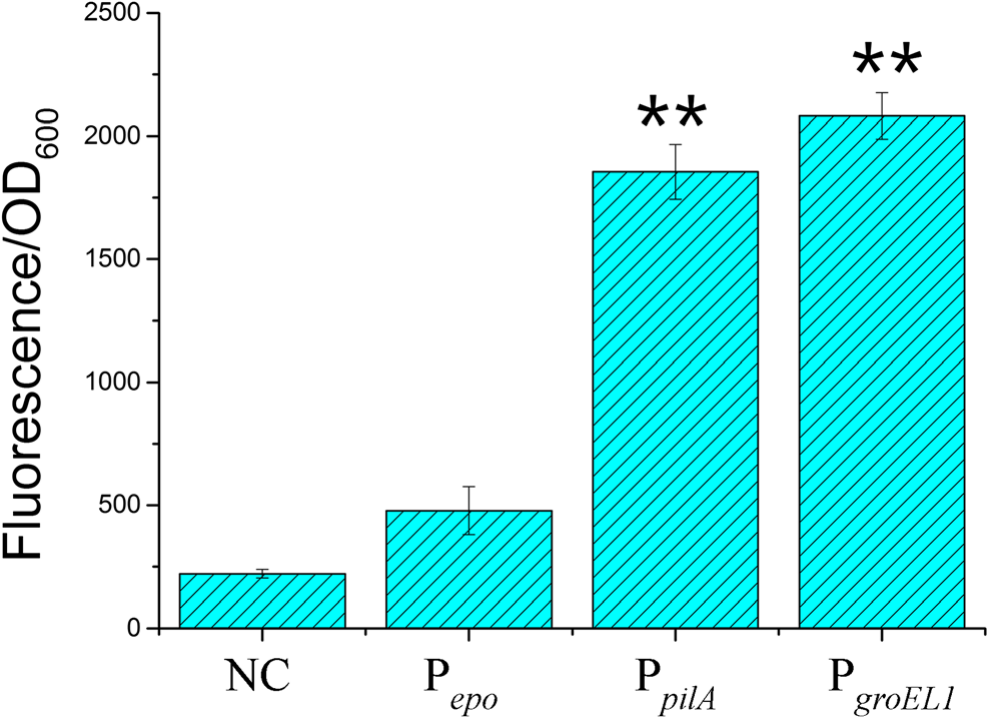
Fluorescence intensity assay of different promoters in *E. coli* after 24 h of incubation. NC, negative control. The error bars represent the standard deviation of three independent experiments. *p value < 0.05; **p value < 0.01

We assayed the transcriptional capacity of these promoters in *E. coli* by using the gene of green fluorescent protein (*gfp*) as the reporter. The *gfp* genes driven by the above three promoters were constructed into the pSWU19 plasmid, respectively (the diagrammatic sketch for the construction is shown in Supplementary Fig. S1). The plasmid pSWU19-*gfp*, which has no promoter upstream the reporter gene, was used as the negative control. We transformed these plasmids separately into *E. coli* and analyzed the cellular fluorescence intensities. Figure 2 shows the fluorescence intensity (the ratio of fluorescence and OD_600_ values) in *E. coli* after 24 h of incubation in LB medium. The *E. coli* cells with the P_*pilA*_- and P_*groEL1*_-controlled *gfp* genes had 6.4-fold and 7.2-fold higher of the fluorescence intensity than the cells with *gfp* controlled by P_*epo*_ did, respectively (the multiples were estimated after subtracting the value of the negative reference; *t* - test, *p* value < 0.01).

### Substitution of the efficient promoters decreases the epothilone production in *M. xanthus*

**Fig. 3:**
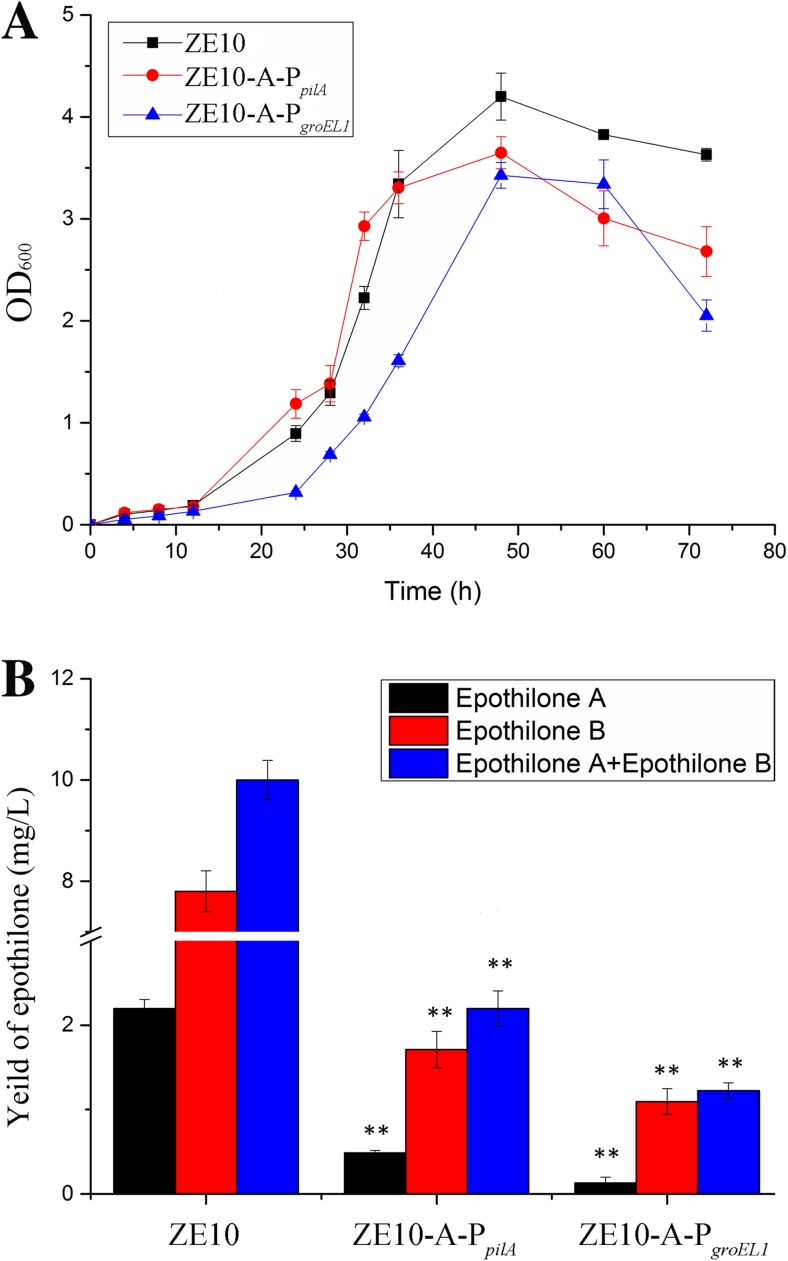
**a** The growth curves of ZE10 and substitution mutants. **b** The epothilones production of ZE10 and substitution mutants. The error bars represent the standard deviation of three independent experiments. **p value < 0.01

We substituted the original promoter P_*epo*_ with P_*pilA*_ or P_*groEL1*_ in *M. xanthus* ZE10, an epothilone-producing strain constructed in our previous work (Zhu et al. 2015b). The substitution vectors were constructed on the basis of the pBJ113 plasmid and transformed by electroporation into ZE10. After two rounds of homologous recombination, the mutants were screened through the *Km*-*galK* cassette, obtaining ZE10-A-P_*pilA*_ and ZE10-A-P_*groEL1*_, respectively. The diagrammatic sketch for the construction is shown in Fig. S3. We screened the mutant strains by colony PCR with the primer pair of L1-F and R1-R (Fig. S4A, B). In CYE medium, the original strain ZE10 reached the maximal growth (the OD_600_ value) after 48 h of incubation (Fig. 3a). The ZE10-A-P_*pilA*_ mutant had a similar growth curve as ZE10 did during the exponential growth stage, but the growth maximum of the mutant was significantly lower than that of ZE10 (*t* - test, *p* value < 0.05). Comparatively, the growth of ZE10-A-P_*groEL1*_ was significantly inhibited not only in the exponential growth stage but also the later stage. The maximal growth had no significant difference between the mutant strains of ZE10-A-P_*pilA*_ and ZE10-A-P_*groEL1*_ (*t* - test, *p* value = 1.000), both of which were lower than those of ZE10.

We assayed the epothilone production in ZE10, ZE10-A-P_*pilA*_, and ZE10-A-P_*groEL1*_ to determine the influences of promoter substitution. The strains were cultivated in 50 mL of the CMO fermentation medium (the CYE medium supplemented with methyl oleate) (Lau et al. 2002), supplemented with 2% of XAD-16 resin for adsorbing the epothilone products (Gong et al. 2007). After 6 days of shaking fermentation, the resin was harvested, and the adsorbed compounds were extracted with methanol for HPLC analysis to determine the yield of epothilones. Surprisingly, although the selected endogenous promoters P_*pilA*_ and P_*groEL1*_ showed stronger transcriptional activities than those of P_*epo*_ in *E. coli*, the epothilone production controlled by the two promoters was greatly lower than that controlled by P_*epo*_ in ZE10. The yield of epothilones was decreased dramatically in the ZE10-A-P_*pilA*_ and ZE10-A-P_*groEL1*_ mutants (*t* - test, *p* value < 0.01), from 10 mg/L in ZE10 to 1.2 mg/L in ZE10-A-P_*groEL1*_ and 2.2 mg/L in ZE10-A-P_*pilA*_, respectively (Fig. 3b).

### P_*epo*_ and its substitutes are in different transcriptional modes

**Fig. 4:**
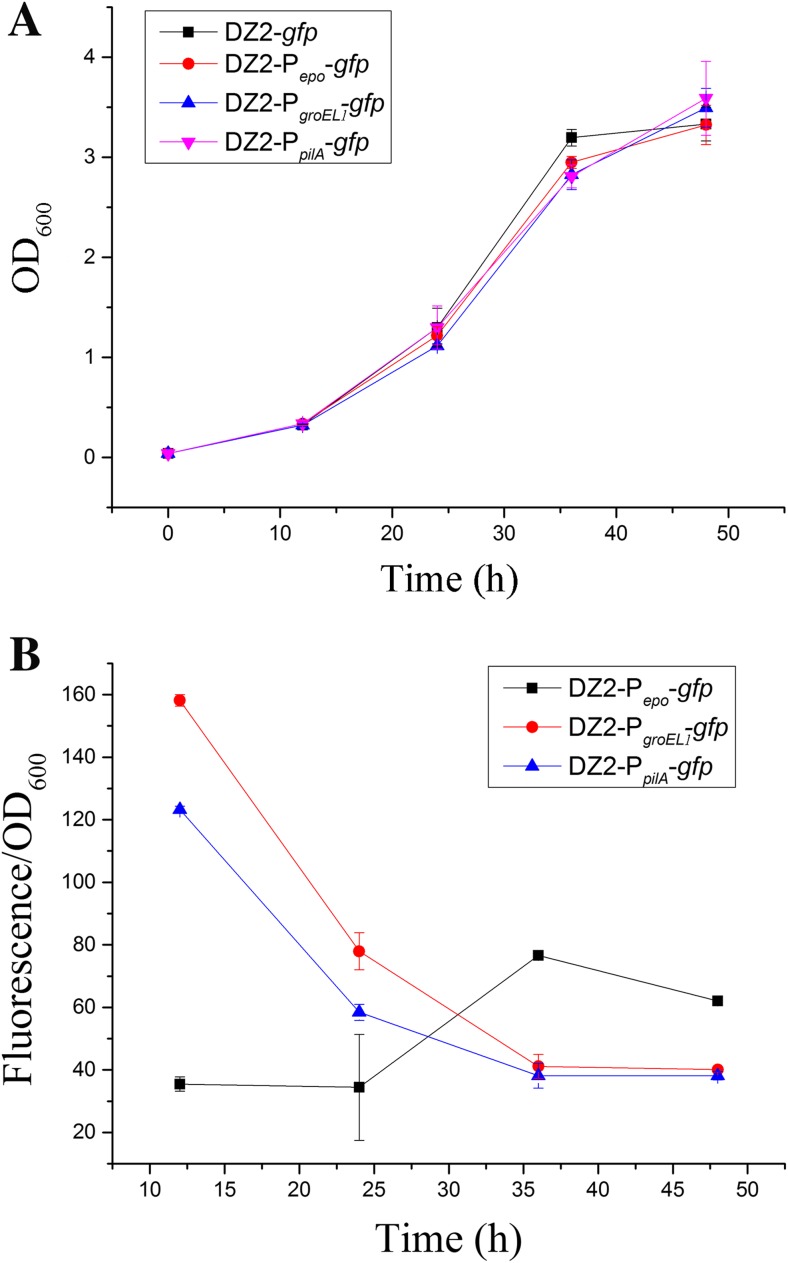
**a** Growth curves of different strains harboring different reporter vectors. **b** The fluorescence intensity assay of different promoters in *M. xanthus* DZ2. The fluorescence intensities shown in the picture were calculated by subtracting the basal value in the DZ2-*gfp* strain (approximately 140 at different incubation time points; *t* - test, *p* value < 0.01). The error bars represent the standard deviation of three independent experiments

In the previous studies, Wu and Kaiser found that the P_*pilA*_ activity was increased to the vegetative level during the first 12 h under the developmental conditions and then decreased rapidly (Wu and Kaiser 1997). Similarly, the P_*groEL1*_ promoter also functioned majorly in the early stage on the TPM development medium (Zhuo et al. 2017). To investigate the involved mechanisms for the epothilone production decrease driven by the P_*pilA*_ and the P_*groEL1*_ promoters, the above constructed pSWU19-based *gfp* reporter vectors, which are able to integrate into the chromosome at the *attB* site in *M. xanthus* (Wu and Kaiser 1995), were introduced into DZ2, respectively, producing the strains of DZ2-*gfp*, DZ2-P_*epo*_-*gfp*, DZ2-P_*groEL1*_-*gfp*, and DZ2-P_*pilA*_-*gfp*. As shown in Fig. 4a, these strains had almost the same growth curve. However, the fluorescence intensities were different in these strains (Fig. 4b). After subtracting the basal value in the DZ2-*gfp* strain (approximately 140 at different incubation time points), the fluorescence intensity of the DZ2-P_*epo*_-*gfp* strain was relatively low but stable during the early growth stage (12–24 h of incubation), then double increased at 36 h, and slightly decreased during the late growth stage (48 h of incubation). Comparatively, the *gfp* reporter controlled by the two endogenous promoters showed different expressional mode: The fluorescence intensities of DZ2-P_*groEL1*_-*gfp* and DZ2-P_*pilA*_-*gfp* were 158 and 123 at 12 h of incubation, which were approximately 3.5-fold and 2.5-fold higher than those of P_*epo*_. Then, the fluorescence intensities of the two strains sharply decreased to about 40 at 36 h and almost unchanged in the following incubation (36–48 h). The fluorescence intensity in the DZ2-P_*epo*_-*gfp* strain during 12–24 h incubation had no difference from that of DZ2-P_*groEL1*_-*gfp* and DZ2-P_*pilA*_-*gfp* during 36–48 h incubation.

The above results showed that P_*epo*_ and its substitutes (P_*pilA*_ and P_*groEL1*_) had different expressional modes. With its own original promoter P_*epo*_, the secondary metabolites epothilones are biosynthesized in the late growth stage and the stable stage, not only in the original producer *S. cellulosum* (Gong et al. 2007) but also in the heterologous *M. xanthus* host (Zhu et al. 2015b). The transcriptional mode of P_*epo*_ is consistent with the epothilone production, i.e., P_*epo*_ guides the epothilone gene expression majorly after the growth of cells, which is suggested to accumulate more epothilone products. In contrast, P_*pilA*_ and P_*groEL1*_ are highly efficient in the growth stage but are markedly decreased in the late growth stage and then unchanged in the stable stage. Accordingly, after swapped with P_*pilA*_ and P_*groEL1*_, the biosynthesis of epothilones followed the transcriptional mode of the promoter substitutes, leading to low yields.

### Tandem-repeat engineering of P_*epo*_ and its effects on transcription

To increase the production ability of epothilones, we delved deeper into the potential of the P_*epo*_ promoter by tandem engineering. In the original promoter of the biosynthetic gene cluster for the production of epothilones, the core region covers the − 290 to − 190 bp upstream of the translation initiation site (+ 1) (Zhu et al. 2013). This region contains two translation initiation sites, and two corresponding − 10 and − 35 regions lie in serial (Fig. 1a). For the recognition of the promoter to initiate transcription, the RNA polymerase binds to promoter and occupies at least 80-bp space (Schmitz and Galas 1979). In the process of RNA polymerases recognition and transcription, the promoter occlusion might appear if there is no sufficient interval space between tandem promoters (Callen et al. 2004; Sneppen et al. 2005). Accordingly, we adopted a 221-bp region covering the − 389 to − 171 bp region as the central promoter fragment, named as cP_*epo*_ (Fig. 1a), for the construction of tandem-repeat promoter. One to three copies of cP_*epo*_ were ligated into the upstream of P_*epo*_ to produce tandem promoters of 1cP_*epo*_-P_*epo*_, 2cP_*epo*_-P_*epo*_, and 3cP_*epo*_-P_*epo*_, respectively (the diagrammatic sketch for the construction is shown in Fig. S2).

**Fig. 5:**
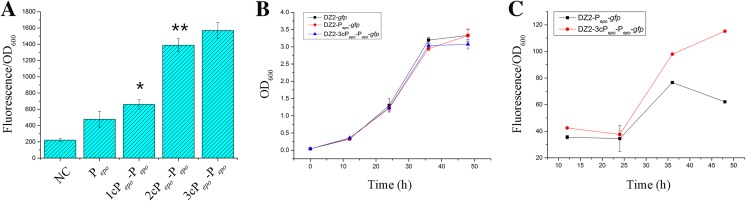
**a** Fluorescence intensity assay of different tandem promoters in *E. coli*. **b** Growth curves of mutant strains harboring different reporter vectors. **c** Fluorescence intensity assay of P_*epo*_ and tandem promoters in *M. xanthus*. The error bars represent the standard deviation of three independent experiments. *p value < 0.05; **p value < 0.01

We firstly monitored the fluorescence intensity of GFP controlled by the tandem engineered promoters in *E. coli*. The fluorescence intensity value was increased by 71% with one cP_*epo*_ stringed upstream P_*epo*_ (*t* - test, *p* value < 0.05), but by 3.5-fold when 2cP_*epo*_ was added (*t* - test, *p* value < 0.01) (Fig. 5a, the multiples were estimated after subtracting the value of negative reference). However, when the cP_*epo*_ number increased to three, the fluorescence intensity was slightly increased relative to that of 2cP_*epo*_-P_*epo*_ (*t* - test, *p* value > 0.05). The result suggested that the transcriptional strength can be enhanced by increasing the tandem repeat of cP_*epo*_, but with a limitation.

Since the three copies of tandem-repeat promoter (3cP_*epo*_-P_*epo*_) showed the strongest fluorescence intensity of GFP in *E. coli*, we further transformed the promoter vector by electroporation into DZ2, producing DZ2-3cP_*epo*_-P_*epo*_-*gfp*, and assayed the expression pattern of the 3cP_*epo*_-P_*epo*_ tandem promoter in *M. xanthus* DZ2. The DZ2-P_*epo*_-*gfp* and DZ2-3cP_*epo*_-P_*epo*_-*gfp* strains exhibited a similar growth curve (Fig. 5b). During the early growth stage in CMO medium (12–24 h of incubation), the P_*epo*_ and 3cP_*epo*_-P_*epo*_ were both in low activity, and 3cP_*epo*_-P_*epo*_ showed little advantage (*t* - test*, p* value > 0.05) (Fig. 5c). During the incubation period of 24–36 h, P_*epo*_ and 3cP_*epo*_-P_*epo*_ both showed increasing activity, and the fluorescence intensity of 3cP_*epo*_-P_*epo*_ was about 28% (36 h of incubation) and 86% (48 h of incubation) higher than that of P_*epo*_ (*t* - test, *p* value < 0.01). Thus, compared with P_*epo*_, the 3cP_*epo*_-P_*epo*_ promoter expressed the *gfp* gene in a similar temporal mode as P_*epo*_ did but appeared to be at a higher level.

### Tandem-repeat promoter increases the epothilone production in *M. xanthus*

**Fig. 6:**
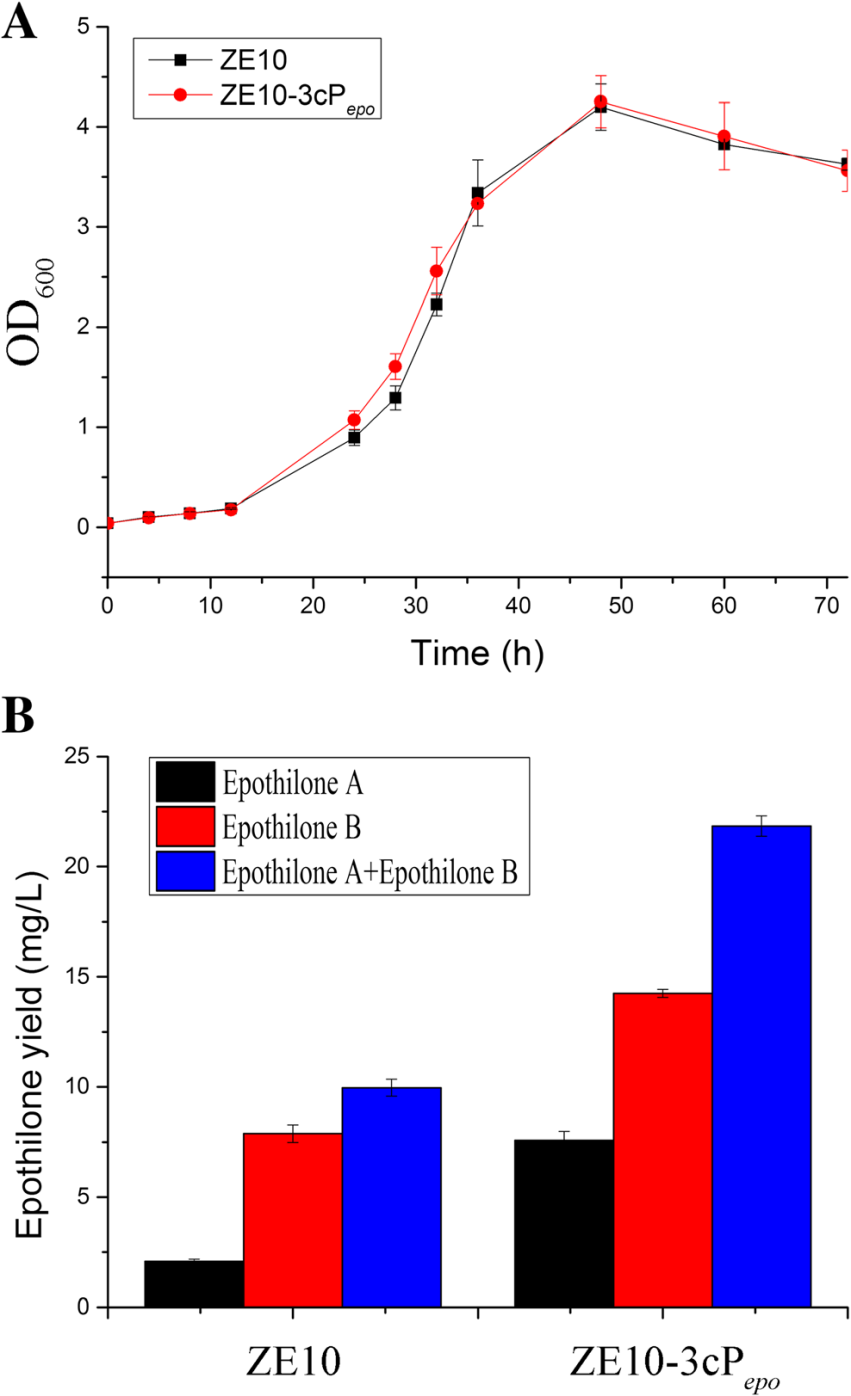
**a** The growth curves of ZE10 and ZE10-3cP*epo*. **b** The epothilone production of ZE10 and ZE10-3cP*epo*. The error bars represent the standard deviation of three independent experiments

We assayed the influence of the 3cP_*epo*_-P_*epo*_ promoter on the production of epothilones in ZE 10. The 3cP_*epo*_ promoter was integrated to the upstream of the original promoter P_*epo*_ in ZE10 to produce ZE10-3cP_*epo*_ strain, in which the epothilone gene cluster was expressed under the control of 3cP_*epo*_-P_*epo*_ (the diagrammatic sketch for the construction is shown in Fig. S5). The ZE10-3cP_*epo*_ strains were screened by colony PCR with primer L2-F and R2-R (Fig. S4C). Cultivated in the CYE medium, the ZE10 and ZE10-3cP_*epo*_ strains showed a similar growth curve (Fig. 6a). Then, the two strains were fermented and the epothilone yields were measured by HPLC. The results showed that the yields of epothilones A and B in ZE10-3cP_*epo*_ increased by 245% and 82.6%, respectively, relative to that of ZE10, and the total production was increased from 10 mg/L in ZE10 to 21.8 mg/L in ZE10-3cP_*epo*_ (Fig. 6b). These results suggested that the tandem repeat promoter transcribed the whole epothilone gene cluster in a similar temporal mode with P_*epo*_, but at an obviously higher level in promoting the epothilone production. This is the first report to construct tandem promoter for the biosynthesis of secondary metabolites. Our result showed that such a tandem engineered promoter is also able to improve the expression of big gene cluster of secondary metabolites.

### Promoters with different modes affected the expressions of the epothilone biosynthetic genes

**Fig. 7:**
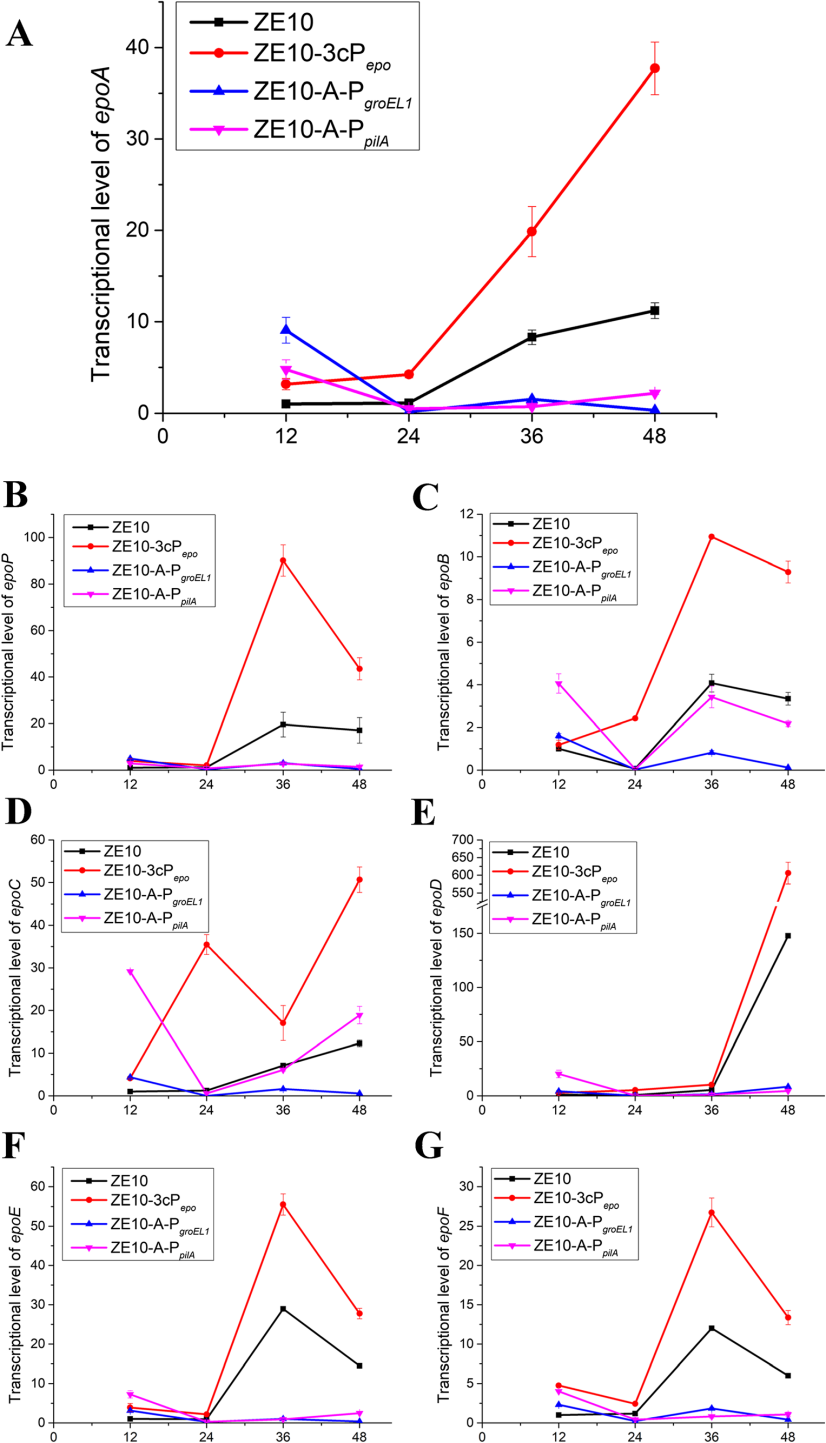
Transcriptional analysis of the epothilone biosynthetic genes in ZE10 and different mutants. **a**–**g** The transcriptional analysis of the epothilone genes *epoA*, *epoP*, *epoB*, *epoC*, *epoD*, *epoE*, and *epoF* in turn. The transcription of each gene in ZE10 at 12 h of incubation was set as 1, respectively. The error bars represent the standard deviation of three independent experiments

To investigate the influence of different promoters on the expression of epothilone biosynthetic genes, we analyzed the transcriptional levels of the epothilone gene cluster in ZE10 and different mutants by using the quantitative real-time polymerase chain reaction (RT-qPCR). Epothilone gene cluster is composed of seven open reading frames (ORFs), spanning about 56 kb in size (*epoA*-*epoF* in Fig. 1a) (Julien et al. 2000; Molnar et al. 2000). The *epoA* gene downstream the promoter was singled out to demonstrate the temporal trend of transcriptional variation driven by different promoters. During the 12–24 h incubation time, *epoA* was transcribed at a relative low but stable level in ZE10 (Fig. 7a). With the increase of incubation time, the *epoA* transcription in ZE10 increased approximately ten times during the stage from 24 to 48 h, just as we reported previously (Yue et al. 2017). Comparatively, the transcriptional levels of *epoA* in ZE10-A-P_*pilA*_ and ZE10-A-P_*groEL1*_ were both higher than that in ZE10 at the 12 h of incubation (*t* - test, *p* value < 0.01) but then decreased dramatically and kept at the low level during the following incubation time. Notably, the transcriptional level of *epoA* in ZE10-3cP_*epo*_ was much higher than that in ZE10 at each of the incubation time points. For example, the transcriptions of *epoA* were increased by 2.2-fold at 12 h of incubation and 2.4-fold at 48 h of incubation, respectively.

The transcriptional levels of each epothilone biosynthetic genes markedly varied in ZE10, which was consistent with our previous reports (Fig. S6) (Yue et al. 2017; Zhu et al. 2015b). The transcriptional trends of different genes in different strains are shown in Fig. 7b–g (the transcription of each gene in ZE10 at 12 h of incubation was set as 1). P_*epo*_ and 3cP_*epo*_-P_*epo*_ expressed all the epothilone genes in a similar pattern, and the tandem engineered promoter worked with more than double efficiency, not only for the front but also the hinder genes. In ZE10 and ZE10-3cP_*epo*_, the transcription level of *epoA* reached to the highest at 48 h of incubation, while the downstream genes showed a trend of ascending followed by leveling off or declining at 48 h of incubation (Fig. 7b, c, f, g), except for *epoC* and *epoD*. The transcriptions of *epoC* and *epoD* were further improved from 36 h of incubation to 48 h of incubation (Fig. 7d, e). The transcriptions of *epoB* (Fig. 7c) and *epoC* (Fig. 7d) in ZE10-A-P_*pilA*_ and ZE10-A-P_*groEL1*_ decreased from 12 h of incubation to 24 h of incubation but turned to increase in the following incubation. However, such variation trend was not found in the transcription of *epoD* in the substitution mutants. The varied expressions and efficiencies by promoters probably reflected that some extra internal promoters in the gene cluster also functioned for the transcriptions of the big gene cluster.

## Discussion

Promoters play a central role in the regulation of gene expression in bacterial and the efficient expression of target genes in heterologous hosts relies on the rational choice of the adequate promoter system (Browning and Busby 2016; Terpe 2006). Aiming to improve the yield of target products, researchers tried to optimize the promoters by random mutation or directional genetic modification and then expressed target genes with appropriate promoter. Without doubt, randomness of mutation renders the screening of promoters with proper strength a time-consuming process. The directional genetic modification is usually based on detailed understanding of the promoters’ organization structure. Substitution of native promoter with desired promoter relies on abundant known promoters workable in specific host. In *E. coli*, a total of 100 stationary-phase promoters have been characterized (Shimada et al. 2004). Similarly, 14 constitutive promoters in *Saccharomyces cerevisiae* (Zhu et al. 2015a) and 32 putative strong promoters in *Streptomyces* (Luo et al. 2015) were also reported. However, limited well-characterized native promoter is available in *M. xanthus*. In this study, we swapped the original promoter of the epothilone biosynthetic gene cluster with two endogenous strong promoters. Decreased production of epothilones in the substitution mutants indicates that the promoters that are efficient in the growth stage are not suitable in the promoter substitution for the production of secondary metabolites.

Tandem promoter, composed of more than one promoter fragments stringed together, exists widely in prokaryotes. For example, all of the *rrn* operons in *E. coli* are transcribed under tandem promoters (Condon et al. 1995; Mackie and Parsons 1983; Young and Steitz 1979). The transcription of *gal* operon in *E. coli* is driven by two overlapped promoters, which respond differently to cyclic AMP (Irani et al. 1989). In *Chlamydia*, some genes are transcribed by tandem promoters, which allow genes to be regulated by multiple transcriptional mechanisms or in different temporal patterns (Rosario and Tan 2015; Shen et al. 2000). In fact, the original promoter of the epothilone biosynthetic gene cluster is also a tandem promoter with two TSSs (Fig. 1a). Inspired by the natural tandem promoters, many tandem promoters have been created to express target genes in different strains. For examples, tandem promoter P_32_-P_*lacA*_ was used to increase the expression of the staphylokinase variant gene in *Lactococcus lactis* (Wei et al. 2002). The constructed P_*amyQ*_-P_*cry3A*_ promoter increased the expression of *aprL* encoding the subtilisin to at least 5-fold when compared with the P_*amyQ*_ or P_*cry3A*_ alone (Widner et al. 2000). P_*LacZ*_-P_*cryIVB*_ and P_*S*_-P_*R*_ tandem promoters remarkably enhanced the mosquitocidal *cryIVB* gene expression and the organophosphorus hydrolase production in *cyanobacteria* (Chungjatupornchai and Fa-Aroonsawat 2014; Soltes-Rak et al. 1993). The tandem promoter engineering was also used in *M. xanthus* for the expression of the *lacl* gene (Vassallo et al. 2017). Notably, almost all of the reported tandem promoters were used to express single small genes (about 1 kb, such as *gfp* gene, *aprL* gene, *cryIVB* gene and so on) or small gene cluster (less than 5 kb, *phaCAB* operon). Few efforts have been made to evaluate the effect of promoters arranged in tandem on the transcriptions of large gene clusters. It is reported that the transcriptional strength reached almost the maximum if the tandem repetitive number of the core-*tac*-promoter (41 bp) increased to five, and more core-*tac*-promoters did not further enhance the transcriptional activity (Li et al. 2012). Similarly, we constructed stronger tandem promoters successfully with cP_*epo*_ in our study. We selected long core promoter region (221 bp) of P_*epo*_ to evade possible promoter occlusion (Callen et al. 2004; Sneppen et al. 2005); the transcription strength of tandem promoter, however, reached close to the maximum when two cP_*epo*_ were stringed upstream P_*epo*_.

As potent anticancer drugs, epothilones biosynthetic genes have been expressed heterologously in different hosts to optimize production or to generate new derivatives. According to the reported studies, *M. xanthus* exhibits as a suitable host for the epothilone productivity. However, lack of efficient promoters hinders the further improvement of epothilone production in *M. xanthus*. We determined that the tandem engineering of P_*epo*_ increased the transcriptional level of epothilone genes by about 2-fold and the epothilones yield was improved by 1.8-fold. We believe that there is a room for further improvement if more suitable promoters are available. Additionally, *epoP*, *epoC*, and *epoD* were transcribed at lower levels relative to that of other epothilone genes (Fig. S6), which might be the rate-determining step in epothilone synthesis. It is worthwhile to identify, characterize, and engineer the possible internal promoters for the epothilone production.

## Electronic supplementary material

ESM 1 (PDF 1037 kb)

## References

[CR1] Alper H, Fischer C, Nevoigt E, Stephanopoulos G (2005). Tuning genetic control through promoter engineering. Proc Natl Acad Sci U S A.

[CR2] Andersen JB, Sternberg C, Poulsen LK, Bjorn SP, Givskov M, Molin S (1998). New unstable variants of green fluorescent protein for studies of transient gene expression in bacteria. Appl Environ Microbiol.

[CR3] Bian X, Tang B, Yu Y, Tu Q, Gross F, Wang H, Li A, Fu J, Shen Y, Li YZ, Stewart AF, Zhao G, Ding X, Muller R, Zhang Y (2017). Heterologous production and yield improvement of epothilones in *Burkholderiales* strain DSM 7029. ACS Chem Biol.

[CR4] Bollag DM, McQueney PA, Zhu J, Hensens O, Koupal L, Liesch J, Goetz M, Lazarides E, Woods CM (1995). Epothilones, a new class of microtubule-stabilizing agents with a taxol-like mechanism of action. Cancer Res.

[CR5] Brogdon CF, Lee FY, Canetta RM (2014). Development of other microtubule-stabilizer families: the epothilones and their derivatives. Anti-Cancer Drugs.

[CR6] Browning DF, Busby SJ (2004). The regulation of bacterial transcription initiation. Nat Rev Microbiol.

[CR7] Browning DF, Busby SJ (2016). Local and global regulation of transcription initiation in bacteria. Nat Rev Microbiol.

[CR8] Callen BP, Shearwin KE, Egan JB (2004). Transcriptional interference between convergent promoters caused by elongation over the promoter. Mol Cell.

[CR9] Chungjatupornchai W, Fa-Aroonsawat S (2014). The rRNA promoter as a tool for the improved expression of heterologous genes in *cyanobacteria*. Microbiol Res.

[CR10] Condon C, Squires C, Squires CL (1995). Control of rRNA transcription in *Escherichia coli*. Microbiol Rev.

[CR11] Craig L, Pique ME, Tainer JA (2004). Type IV pilus structure and bacterial pathogenicity. Nat Rev Microbiol.

[CR12] Fu J, Wenzel SC, Perlova O, Wang J, Gross F, Tang Z, Yin Y, Stewart AF, Muller R, Zhang Y (2008). Efficient transfer of two large secondary metabolite pathway gene clusters into heterologous hosts by transposition. Nucleic Acids Res.

[CR13] Gerth K, Bedorf N, Hofle G, Irschik H, Reichenbach H (1996). Epothilons A and B: antifungal and cytotoxic compounds from *Sorangium cellulosum* (*Myxobacteria*). Production, physico-chemical and biological properties. J Antibiot (Tokyo).

[CR14] Gong GL, Sun X, Liu XL, Hu W, Cao WR, Liu H, Liu WF, Li YZ (2007). Mutation and a high-throughput screening method for improving the production of Epothilones of *Sorangium*. J Ind Microbiol Biotechnol.

[CR15] Han K, Li ZF, Peng R, Zhu LP, Zhou T, Wang LG, Li SG, Zhang XB, Hu W, Wu ZH, Qin N, Li YZ (2013). Extraordinary expansion of a *Sorangium cellulosum* genome from an alkaline milieu. Sci Rep.

[CR16] Huang H, Menefee M, Edgerly M, Zhuang S, Kotz H, Poruchynsky M, Huff LM, Bates S, Fojo T (2010). A phase II clinical trial of ixabepilone (Ixempra; BMS-247550; NSC 710428), an epothilone B analog, in patients with metastatic renal cell carcinoma. Clin Cancer Res.

[CR17] Irani M, Musso R, Adhya S (1989). Cyclic-AMP-dependent switch in initiation of transcription from the two promoters of the *Escherichia coli* gal operon: identification and assay of 5′-triphosphate ends of mRNA by GTP:RNA guanyltransferase. J Bacteriol.

[CR18] Jakovljevic V, Leonardy S, Hoppert M, Sogaard-Andersen L (2008). PilB and PilT are ATPases acting antagonistically in type IV pilus function in *Myxococcus xanthus*. J Bacteriol.

[CR19] Julien B, Shah S (2002). Heterologous expression of Epothilone biosynthetic genes in *Myxococcus xanthus*. Antimicrob Agents Chemother.

[CR20] Julien B, Shah S, Ziermann R, Goldman R, Katz L, Khosla C (2000). Isolation and characterization of the epothilone biosynthetic gene cluster from *Sorangium cellulosum*. Gene.

[CR21] Kerner MJ, Naylor DJ, Ishihama Y, Maier T, Chang HC, Stines AP, Georgopoulos C, Frishman D, Hayer-Hartl M, Mann M, Hartl FU (2005). Proteome-wide analysis of chaperonin-dependent protein folding in *Escherichia coli*. Cell.

[CR22] Lau J, Frykman S, Regentin R, Ou S, Tsuruta H, Licari P (2002). Optimizing the heterologous production of epothilone D in *Myxococcus xanthus*. Biotechnol Bioeng.

[CR23] Li MJ, Wang JS, Geng YP, Li YK, Wang Q, Liang QF, Qi QS (2012). A strategy of gene overexpression based on tandem repetitive promoters in *Escherichia coli*. Microb Cell Fact.

[CR24] Liu T, Wang TH, Li X, Liu X (2008). Improved heterologous gene expression in *Trichoderma reesei* by cellobiohydrolase I gene (cbh1) promoter optimization. Acta Biochim Biophys Sin.

[CR25] Luo YZ, Zhang L, Barton KW, Zhao HM (2015). Systematic identification of a panel of strong constitutive promoters from *Streptomyces albus*. ACS Synth Biol.

[CR26] Mackie GA, Parsons GD (1983). Tandem promoters in the gene for ribosomal protein S20. J Biol Chem.

[CR27] Molnar I, Schupp T, Ono M, Zirkle RE, Milnamow M, Nowak-Thompson B, Engel N, Toupet C, Stratmann A, Cyr DD, Gorlach J, Mayo JM, Hu A, Goff S, Schmid J, Ligon JM (2000). The biosynthetic gene cluster for the microtubule-stabilizing agents epothilones A and B from *Sorangium cellulosum* So ce90. Chem Biol.

[CR28] Müller R (2009). Biosynthesis and heterologous production of epothilones. Fortschr Chem Org Naturst.

[CR29] Müller S, Willett JW, Bahr SM, Darnell CL, Hummels KR, Dong CK, Vlamakis HC, Kirby JR (2013). Draft genome sequence of *Myxococcus xanthus* wild-type strain DZ2, a model organism for predation and development. Genome Announc.

[CR30] Mutka SC, Carney JR, Liu Y, Kennedy J (2006). Heterologous production of epothilone C and D in *Escherichia coli*. Biochemistry.

[CR31] Osswald C, Zipf G, Schmidt G, Maier J, Bernauer HS, Muller R, Wenzel SC (2014). Modular construction of a functional artificial epothilone polyketide pathway. ACS Synth Biol.

[CR32] Park SR, Park JW, Jung WS, Han AR, Ban YH, Kim EJ, Sohng JK, Sim SJ, Yoon YJ (2008). Heterologous production of epothilones B and D in *Streptomyces venezuelae*. Appl Microbiol Biotechnol.

[CR33] Peng R, Chen JH, Feng WW, Zhang Z, Yin J, Li ZS, Li YZ (2017). Error-prone DnaE2 balances the genome mutation rates in *Myxococcus xanthus* DK1622. Front Microbiol.

[CR34] Qiu YM, Xiao F, Wei XT, Wen ZY, Chen SW (2014). Improvement of lichenysin production in *Bacillus licheniformis* by replacement of native promoter of lichenysin biosynthesis operon and medium optimization. Appl Microbiol and Biot.

[CR35] Rivera E, Lee J, Davies A (2008). Clinical development of ixabepilone and other epothilones in patients with advanced solid tumors. Oncologist.

[CR36] Roche H, Yelle L, Cognetti F, Mauriac L, Bunnell C, Sparano J, Kerbrat P, Delord JP, Vahdat L, Peck R, Lebwohl D, Ezzeddine R, Cure H (2007). Phase II clinical trial of ixabepilone (BMS-247550), an epothilone B analog, as first-line therapy in patients with metastatic breast cancer previously treated with anthracycline chemotherapy. J Clin Oncol.

[CR37] Rosario CJ, Tan M (2015). Regulation of *chlamydia* gene expression by tandem promoters with different temporal patterns. J Bacteriol.

[CR38] Roy AL, Singer DS (2015). Core promoters in transcription: old problem, new insights. Trends Biochem Sci.

[CR39] Schmitz A, Galas DJ (1979). Interaction of RNA polymerase and lac repressor with the lac control region. Nucleic Acids Res.

[CR40] Shen L, Shi Y, Douglas AL, Hatch TP, O'Connell CM, Chen JM, Zhang YX (2000). Identification and characterization of promoters regulating tuf expression in *Chlamydia trachomatis* serovar F. Arch Biochem Biophys.

[CR41] Shimada T, Makinoshima H, Ogawa Y, Miki T, Maeda M, Ishihama A (2004). Classification and strength measurement of stationary-phase promoters by use of a newly developed promoter cloning vector. J Bacteriol.

[CR42] Sneppen K, Dodd IB, Shearwin KE, Palmer AC, Schubert RA, Callen BP, Egan JB (2005). A mathematical model for transcriptional interference by RNA polymerase traffic in *Escherichia coli*. J Mol Biol.

[CR43] Soltes-Rak E, Kushner DJ, Williams DD, Coleman JR (1993). Effect of promoter modification on mosquitocidal cryIVB gene expression in *Synechococcus* sp. strain PCC 7942. Appl Environ Microbiol.

[CR44] Splinter E, de Laat W (2011). The complex transcription regulatory landscape of our genome: control in three dimensions. EMBO J.

[CR45] Tang L, Shah S, Chung L, Carney J, Katz L, Khosla C, Julien B (2000). Cloning and heterologous expression of the epothilone gene cluster. Science.

[CR46] Terpe K (2006). Overview of bacterial expression systems for heterologous protein production: from molecular and biochemical fundamentals to commercial systems. Appl Microbiol Biotechnol.

[CR47] Vassallo CM, Cao PB, Conklin A, Finkelstein H, Heyer CS, Wall D (2017) Infectious polymorphic toxins delivered by outer membrane exchange discriminate kin in *myxobacteria*. 6. 10.7554/eLife.29397 PMC5562445 28820387

[CR48] Vichai V, Kirtikara K (2006). Sulforhodamine B colorimetric assay for cytotoxicity screening. Nat Protoc.

[CR49] Wang Y, Zhang WY, Zhang Z, Li J, Li ZF, Tan ZG, Zhang TT, Wu ZH, Liu H, Li YZ (2013). Mechanisms involved in the functional divergence of duplicated groEL chaperonins in *Myxococcus xanthus* DK1622. PLoS Genet.

[CR50] Wei WZ, Xiang H, Tan HR (2002). Two tandem promoters to increase gene expression in *Lactococcus lactis*. Biotechnol Lett.

[CR51] Widner B, Thomas M, Sternberg D, Lammon D, Behr R, Sloma A (2000). Development of marker-free strains of *Bacillus subtilis* capable of secreting high levels of industrial enzymes. J Ind Microbiol Biotechnol.

[CR52] Wu SS, Kaiser D (1995). Genetic and functional evidence that type IV pili are required for social gliding motility in *Myxococcus xanthus*. Mol Microbiol.

[CR53] Wu SS, Kaiser D (1997). Regulation of expression of the pilA gene in *Myxococcus xanthus*. J Bacteriol.

[CR54] Young RA, Steitz JA (1979). Tandem promoters direct *E. coli* ribosomal RNA synthesis. Cell.

[CR55] Yue XJ, Cui XW, Zhang Z, Peng R, Zhang P, Li ZF, Li YZ (2017). A bacterial negative transcription regulator binding on an inverted repeat in the promoter for epothilone biosynthesis. Microb Cell Factories.

[CR56] Zhu LP, Li ZF, Sun X, Li SG, Li YZ (2013). Characteristics and activity analysis of epothilone operon promoters from *Sorangium cellulosum* strains in *Escherichia coli*. Appl Microbiol Biotechnol.

[CR57] Zhu Duolong, Liu Fulu, Xu Haijin, Bai Yanling, Zhang Xiuming, Saris Per Erik Joakim, Qiao Mingqiang (2015). Isolation of strong constitutive promoters fromLactococcus lactissubsp.lactisN8. FEMS Microbiology Letters.

[CR58] Zhu LP, Yue XJ, Han K, Li ZF, Zheng LS, Yi XN, Wang HL, Zhang YM, Li YZ (2015). Allopatric integrations selectively change host transcriptomes, leading to varied expression efficiencies of exotic genes in *Myxococcus xanthus*. Microb Cell Factories.

[CR59] Zhuo L, Wang Y, Zhang Z, Li J, Zhang XH, Li YZ (2017). *Myxococcus xanthus* DK1622 coordinates expressions of the duplicate *groEL* and single *groES* genes for synergistic functions of groELs and groES. Front Microbiol.

