# Supplementary material for: Effects of transcriptional mode on promoter substitution and tandem engineering for the production of epothilones in Myxococcus xanthus
Source: Appl Microbiol Biotechnol. 2018 Apr 28;102(13):5599–610. doi: 10.1007/s00253-018-9023-4 (PMC5999154; doi:10.1007/s00253-018-9023-4)
Supplement: Supplementary file 1 — (PDF 1037 kb) [file 253_2018_9023_MOESM1_ESM.pdf]

Supplementary Material

Journal: *Applied Microbiology and Biotechnology*

Tittle: Effects of transcriptional mode on promoter substitution and tandem engineering for the production of epothilones in *Myxococcus xanthus*

Authors: Xin-jing Yue[§], Xiao-wen Cui[§], Zheng Zhang, Wei-feng Hu, Zhi-feng Li, You-ming Zhang, Yue-zhong Li*

[§] These authors contribute equally to this paper. *The corresponding author.

State Key Laboratory of Microbial Technology, School of Life Science, Shandong University, Jinan 250100, China

Authors' email addresses:

Xin-jing Yue, xinjing92@126.com

Xiao-wen Cui, cuixw93@163.com

Zheng Zhang, zhzhang.sdu@gmail.com

Wei-feng Hu, weifenghu94@163.com

Zhi-feng Li, lizhifeng@sdu.edu.cn

You-ming Zhang, zhangyouming@sdu.edu.cn

Yue-zhong Li, llilab@sdu.edu.cn,
Tel: +86-531-88564288; Fax: +86-531-88564288

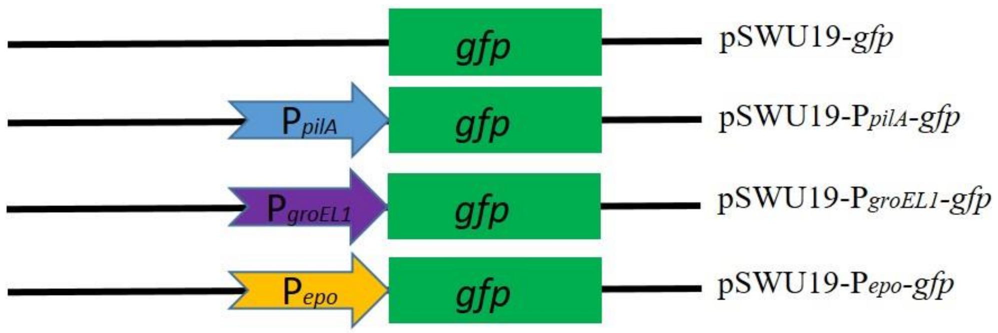

Fig S1. Diagrammatic sketch for the construction of *gfp* reporter vectors

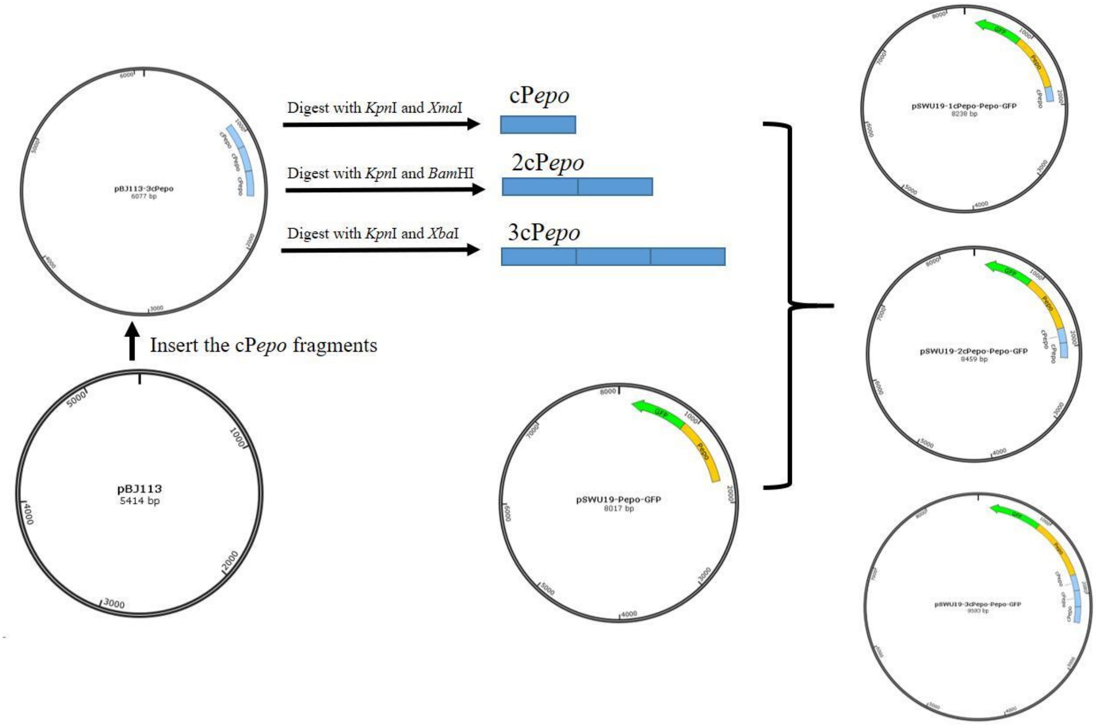

Fig S2. Diagrammatic sketch for the construction of tandem promoter clusters and corresponding reporter vectors

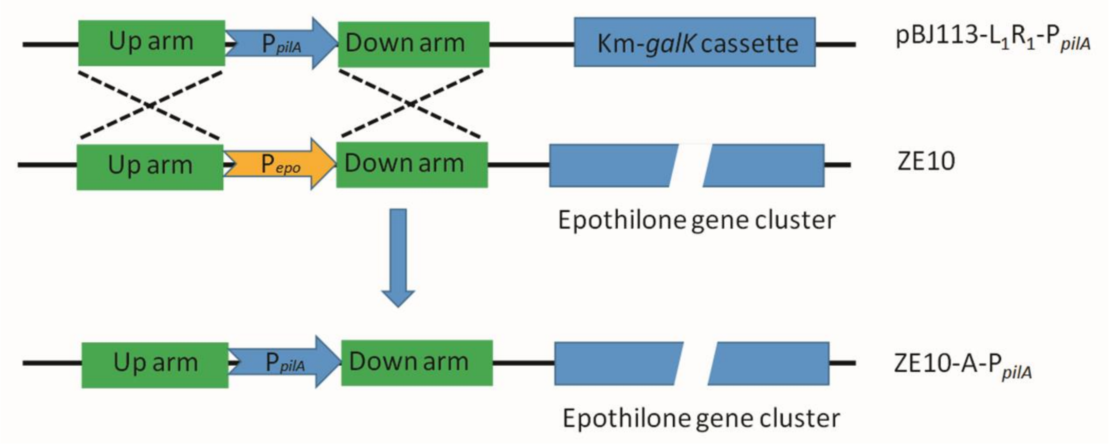

Fig S3. Diagrammatic sketch for the construction of substitution vectors and substitution mutants. Up arm and down arm represent the homologous arms for homologous recombination; Km-*galK* represent the selection markers (kanamycin resistant gene and galactokinase gene).

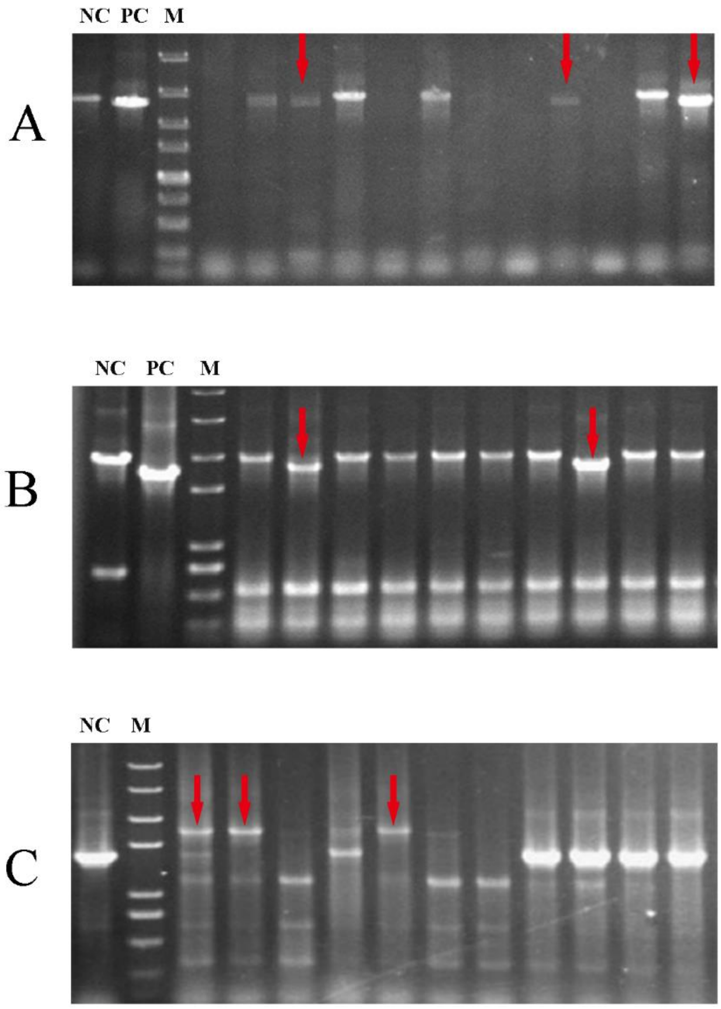

Fig S4. Screening of the promoter manipulation mutant strains by colony PCR. The lanes marked with red arrow were the positive mutant strains. (A) Screening of ZE10-A-P*pilA*. NC, the DZ2 genome was used as template; PC, plasmid pBJ113-L_2R_2-P*pilA* was used as template; M, the DL5000 DNA Marker (from top to bottom, 5,000 bp, 3,000 bp, 2,000 bp, 1,500 bp, 1,000 bp, 750 bp, 500 bp, 250 bp, and 100 bp). (B) Screening of ZE10-A-P*groEL1*. NC, the DZ2 genome was used as template; PC, plasmid pBJ113-L_2R_2-P*groEL1* was used as template; M, the Trans 2K Plus II DNA Marker (from top to bottom, 8,000 bp, 5,000 bp, 3,000 bp, 2,000 bp, 1,000 bp, 750 bp, 500 bp, 250 bp, and 100 bp). (C) Screening of ZE10-3cP*epo*. NC, the ZE10 genome was used as template; M, the Trans 2K Plus II DNA Marker.

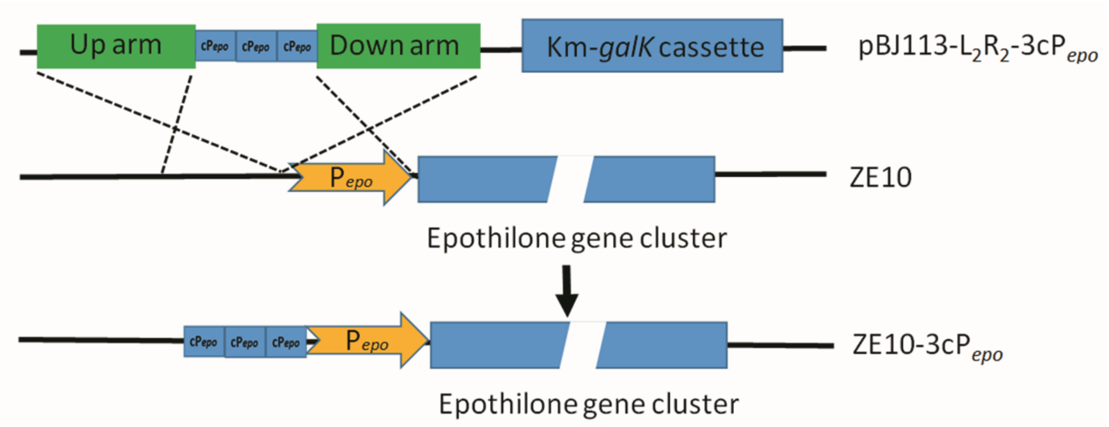

Fig S5. Diagrammatic sketch for the construction of mutants with tandem promoters. Up arm and down arm represent the homologous arm for homologous recombination; Km-*galK* represent the selection markers (kanamycin resistant gene and galactokinase gene).

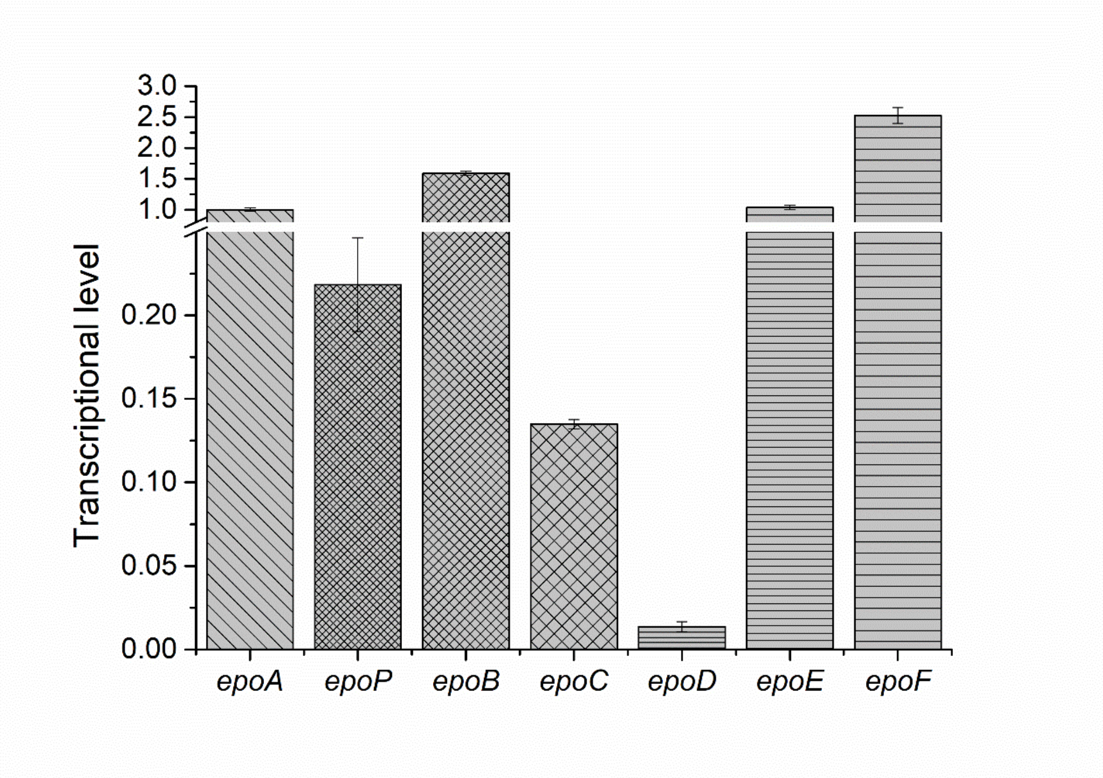

Fig S6. The relative transcriptional levels of epothilone genes in ZE10 at 12 h of incubation. The transcriptional level of *epoA* was set as 1. The error bars represent the standard deviation of three independent experiments.

Table S1. Strains and plasmids used in this study

Strain and plasmid	Relevant characteristics	Source or reference
Strains		
E. coli DH5α	F−, *supE44, ΔlacU169 (ϕ80lacZΔM15), hsdR17, recA1, endA1, gyrA96, thi-1, relA1*	Life Technologies
E. coli DH5α (*λ pir*)	DH5α containing *λ pir* gene	H.B. Kaplan
M. xanthus DZ2	Derived from *M. xanthus* FB (ATCC 19368)	(Müller et al. 2013)
M. xanthus DK 1622	Derived from *M. xanthus* FB (ATCC 19368)	(Wall et al. 1999)
M. xanthus ZE10	A heterologous expressional host of epothilone, derived from *M. xanthus* DZ2	This lab
DZ2-*gfp*	DZ2 with integration of pSWU19-*gfp*	This study
DZ2-P_{pilA}-*gfp*	DZ2 with integration of pSWU19-P_{pilA}-*gfp*	This study
DZ2-P_{groEL}-*gfp*	DZ2 with integration of pSWU19-P_{groEL1}-*gfp*	This study
DZ2-P_{epo}-*gfp*	DZ2 with integration of pSWU19-P_{epo}-*gfp*	This study
DZ2-ncP_{epo}-P_{epo}-*gfp*	DZ2 with integration of pSWU19-ncP_{epo}-P_{epo}-*gfp* (n=1,2,3)	This study
ZE10-3cP_{epo}	ZE10 with 3cP_{epo} inserted upstream of P_{epo}	This study
ZE10-A-P_{pilA}	ZE10 with P_{epo} replaced by P_{pilA}	This study
ZE10-A-P_{groEL1}	ZE10 with P_{epo} replaced by P_{groEL}	This study
Plasmids		
pJBA28	Containing *gfp* gene, Ap^r; Km^r	(Andersen et al. 1998)
p15A-CT-epo	Containing epothilone gene cluster and flanking sequence, $Apra^r$	This lab
pBJ113	Gene replacement vector with KG cassette; Km^r	Z.M. Yang, Virginia Tech
pSWU19	Site specific integration vector with Mx8 attB integration site (Mx8); Km^r	(Wu and Kaiser 1997)
pBJ113-3cP_{epo}	Ligating three tandem promoters to the pBJ113	This study
pBJ113-L_1R_1-3cP_{epo}	Ligating the two homologous arms for insertion to the pBJ113	This study
pBJ113-L_2R_2-P_{pilA}	Ligating the two homologous arms for replacement the pBJ113	This study
pBJ113-L_2R_2 P_{groEL1}	Ligating the two homologous arms for replacement the pBJ113	This study
pSWU19-*gfp*	Ligating *gfp* to the pSWU19	This study
pSWU19-P_{epo}-*gfp*	Ligating P_{epo}-*gfp* fusion fragment to the pSWU19	This study
pSWU19-P_{pilA}-*gfp*	Ligating P_{pilA} fragment to the pSWU19-*gfp*	This study
pSWU19-P_{groEL1}-*gfp*	Ligating P_{groEL} fragment to the pSWU19-*gfp*	This study
pSWU19-ncP_{epo}-P_{epo}-*gfp*	Ligating n cP_{epo} fragment to the pSWU19-P_{epo}-*gfp* (n=1,2,3)	This study

Table S2. Primers used in construction of vectors and mutant strains

Primer name	Sequence (5'-3')	Restriction Site
M13-F	CAGGAAACAGCTATGACC	None
M13-R	TGTAAAACGACGGCCAGT	None
P1-F	GGGGTACCGTCGTAACCGCCCAGCAA	*Kpn*I
P1-R	TCCCCCCGGGGACGGGCACATCCTCAGCG	*Xma*I
P2-F	TCCCCCCGGGGTCGTAACCGCCCAGCAA	*Xma*I
P2-R	CGGGATCCGACGGGCACATCCTCAGCG	*Bam*HI
P3-F	CGGGATCCGTCGTAACCGCCCAGCAA	*Bam*HI
P3-R	CGTCTAGAGACGGGCACATCCTCAGCG	*Xba*I
pE-F	GCTCTAGAGCCTTGGTCATGTGGTGTTCGTGCGTC	*Xba*I
pE-R	AGCTCGGCGCCCTTGCTCACCATGACGGGCACATCCTCAGCG	None
P_{pilA}-F	GGGGTACCCTGGCGAACTACTTCCTGTC	*Kpn*I
P_{pilA}-R	GCTCTAGAGGGGGTCCTCAGAGAAGGTTG	*Xba*I
P_{groEL1}-F	GGGGTACCCCAGCGTCGCTTGACGGGTCG	*Kpn*I
P_{groEL1}-R	GCTCTAGATTGGATGGTTCCTTGAAGGAA	*Xba*I
gfp-F1	ATGGTGAGCAAGGGCGCCGAGCT	None
gfp-R1	CCCAAGCTTTCACTTGTACAGCTCATCCATGCCGTGG	*Hin*dIII
gfp-F2	GCTCTAGAATGGTGAGCAAGGGCGCCGAGCT	*Xba*I
gfp-R2	CCCAAGCTTTCACTTGTACAGCTCATCCATGCCGTGG	*Hin*dIII
pSWU19-F	GCAAGGCGATTAAGTTGGGTA	None
pSWU19-R	GGCTCGTATGTTGTGTGG	None
L1-F	GGAATTCTCGCGACTGGCTCGACAACTT	*Eco*RI
L1-R	GGGGTACCGCGGCGCGGGCTCAATGTCGG	*Kpn*I
R1-F	GCTCTAGAGCCTTGGTCATGTGGTGTTCG	*Xba*I
R1-R	CCCAAGCTTGATCGTCCTCCGGTCGAGCCC	*Hin*dIII
L2-F	GGAATTCTCGCGACTGGCTCGACAACTT	*Eco*RI
L2-R	GGGGTACCGCGGCGCGGGCTCAATGTCG	*Kpn*I
R2-F	GTCTAGAGTGGCGGATCGTCCCATCG	*Xba*I
R2-R	CCCAAGCTTGATCGTCCTCCGGTCGAGCCC	*Hin*dIII
KG-F	GCTCTAGAGCTAGCCGAAATGACCGACCAAGC	None
KG-R	GGTGCCAGTGCGGGAGTTTCG	None
Test-F	CAGTATGCAAAGTTCTGGATCG	None
Test-R	GATCCGCCACGACGGGCACAT	None

Table S3. Primers used in RT-qPCR.

Primer name	Sequence (5'-3')
epoA-F	GCGTTCCACTCACCGCTCAT
epoA-R	GCCTTCCCGCTCAGATTGCT
epoP-F	GCTCAACATAACGCTCTTCAACC
epoP-R	CTGGACCTCGATACCGCTCA
epoB-F	ATGGAAGAACAAGATTCCTC
epoB-R	CTCGGAGAAGCGCTGCACGG
epoC-F	GAAGATGCGGTGAGGTTGGTGG
epoC-R	TCGGACGCTGCGATGGCTAC
epoD-F	GTGACAGACCGAGAAGGAC
epoD-R	CCACGATGGCGATCGGCTCG
epoE-F	GCACCGTTTGCGTTAGTAGGG
epoE-R	GCTTGGCTATTATGTCGGTCTCC
epoF-F	GGAGCAAGCGAATCAGAGTG
epoF-R	CGTGGTATCGGGTGAGGAC
gapA-F	GCCCTGGAAGAGCCTGAACG
gapA-R	TGGAGACGATGTGGTGCTTGG
